# Isolation, Characterization, and Molecular Detection of Porcine Sapelovirus

**DOI:** 10.3390/v14020349

**Published:** 2022-02-08

**Authors:** Yassein M. Ibrahim, Wenli Zhang, Gebremeskel Mamu Werid, He Zhang, Yawen Feng, Yu Pan, Lin Zhang, Changwen Li, Huan Lin, Hongyan Chen, Yue Wang

**Affiliations:** 1 Heilongjiang Provincial Key Laboratory of Laboratory Animal and Comparative Medicine, State Key Laboratory of Veterinary Biotechnology, National Poultry Laboratory Animal Resource Center, Harbin Veterinary Research Institute, Chinese Academy of Agricultural Sciences, Harbin 150069, China; yassin8322@gmail.com (Y.M.I.); zwl5561@163.com (W.Z.); ashenafymamo@gmail.com (G.M.W.); zhanghe3789@163.com (H.Z.); gudaoqiusheng37@163.com (Y.P.); zhanglin19920402@163.com (L.Z.); yuewang_vet2022@163.com (C.L.); yuerdecopy@126.com (H.L.); chenhongyan@caas.cn (H.C.); 2 Laboratory of Inspection and Testing, Hebei Provincial Station of Veterinary Drug and Feed, Shijiazhuang 050000, China; tianehu2022@126.com

**Keywords:** porcine sapelovirus, isolation, characterization, prevalence, monoclonal antibodies, ELISA

## Abstract

Porcine sapelovirus (PSV) is an important emerging pathogen associated with a wide variety of diseases in swine, including acute diarrhoea, respiratory distress, skin lesions, severe neurological disorders, and reproductive failure. Although PSV is widespread, serological assays for field-based epidemiological studies are not yet available. Here, four PSV strains were recovered from diarrheic piglets, and electron microscopy revealed virus particles with a diameter of ~32 nm. Analysis of the entire genome sequence revealed that the genomes of PSV isolates ranged 7569–7572 nucleotides in length. Phylogenetic analysis showed that the isolated viruses were classified together with strains from China. Additionally, monoclonal antibodies for the recombinant PSV-VP1 protein were developed to specifically detect PSV infection in cells, and we demonstrated that isolated PSVs could only replicate in cells of porcine origin. Using recombinant PSV-VP1 protein as the coating antigen, we developed an indirect ELISA for the first time for the detection of PSV antibodies in serum. A total of 516 swine serum samples were tested, and PSV positive rate was 79.3%. The virus isolates, monoclonal antibodies and indirect ELISA developed would be useful for further understanding the pathophysiology of PSV, developing new diagnostic assays, and investigating the epidemiology of the PSV.

## 1. Introduction

*Sapelovirus* is a new genus within the family *Picornaviridae*. Currently, this genus comprises porcine *Sapelovirus* (PSV), simian *Sapelovirus*, avian *Sapelovirus*, and unclassified *Sapelovirus* isolated from bat, marmot, California sea lion, and mouse [1]. The *Sapelovirus* genome is a positive-strand RNA of approximately 7.5 kb in length, with the typical picornavirus genome organization: a 5′ untranslated region (UTR), a single large open reading frame (ORF), a 3′-UTR, and a poly (A) tail. The ORF encodes a single polyprotein that is subsequently cleaved by virus encoding proteases into twelve proteins, including leader protein, four structural proteins (VP1-VP4) and seven nonstructural functional proteins (2A, 2B, 2C, 3A, 3B, 3C, and 3D) [2]. The capsid proteins of *Sapelovirus* are composed of four structural proteins located at the virion surface and exhibit high sequence variability [1]. VP1 protein, the most dominant and variable viral protein, has proven useful in determining genetic relationships among picornaviruses [3,4,5,6].

PSV is transmitted through the fecal-oral route and associated with various symptoms, ranging from asymptomatic to clinical diseases such as respiratory distress, acute diarrhea, skin lesions, severe neurological disorders, and reproductive failure in domestic swine and wild boar [7,8,9,10,11]. Since first reported in UK [12], PSV has been identified in different countries worldwide, with prevalence ranging between 7.1% in India and 71.0% in Hungary [2,13,14,15,16,17,18,19,20,21]. Recent reports have shown that PSV can induce intestinal lesions in experimentally infected piglets [1,8,22]. Previous studies have recorded lethal PSV infections in pigs aged 3 to 12 weeks with neurological problems, diarrhea, and respiratory ailments [7,10,11]. The coinfection of viral diarrhea agents complicates disease detection, control, and prevention. Coinfection of PSV with other enteric pathogens is frequently reported in both symptomatic and asymptomatic pigs [4,10,18,20,22,23,24,25]. Further, the asymptomatic nature of PSV infections and the high coinfection rate cause the symptoms of PSV infection to go unnoticed [24], which poses a considerable risk to swine industries. Because antiviral drug and vaccine are not available yet, early identification and accurate diagnosis play a decisive role in timely containment and control of PSV infection.

Currently, diagnosis of PSV is mainly based on the detection of nucleic acid by PCR and confirmed by virus isolation [1,15,19]. Although detection of PSV by PCR is sensitive, serological diagnosis should be more accurate. However, no specific antibody-based reagent or serological assay is commercially available yet to detect PSV infections. Based on these facts, highly sensitive and effective diagnostic assays are crucial to investigate the epidemiology of PSV. Thus, the objectives of this study were to generate monoclonal antibodies (mAbs), develop an indirect ELISA assay, and explore the prevalence of PSV on swine farms.

## 2. Materials and Methods

### 2.1. Sample Collection

Six fecal specimens were collected from piglets with diarrhea from two farms in Heilongjiang province and submitted to our lab for routine diagnostic purposes. In addition, a total of 291 samples including, fecal, rectal swabs and intestinal contents (Table 1). Furthermore, a total of 516 serum samples were randomly collected for routine monitoring. All samples were stored at −80 °C until use.

### 2.2. Cell Culture

Nineteen cell lines derived from different species were used, including pig (PK15, ST, IPEC-J2), human (293T, Huh7, HepG2, A549, Hela, CaCo2), primate (Marc145, Vero E6), bovine (MDBK), canine (MDCK), feline (CRFK), rabbit (RK13), hamster (BHK21, CHO), duck (DEF), and chicken (DF-1) cell lines. Marc145 cell was cultured in RPMI-1640 medium (Gibco, Beijing, China), DEF and DF-1 cells in Eagle’s Minimum Essential Medium (EMEM, Multicell, Beijing, China), and the remaining cells were cultured in Dulbecco’s modified Eagle’s medium (DMEM, Gibco). All media were supplemented with 10% (*v*/*v*) fetal bovine serum (FBS) (Biological Industries, Beijing, China), 100 U/mL penicillin and 100 U/mL streptomycin.

### 2.3. Virus Isolation

To identify the causative agent of six diarrheic piglets, fecal samples were examined by RT-PCR for swine enteropathogenic viruses, including transmissible gastroenteritis virus (TGEV), porcine epidemic diarrhoea virus (PEDV), porcine deltacoronavirus (PDCoV), rotavirus genogroup A (RVA), porcine sapovirus (PSaV), porcine Kobuvirus (PKV), enterovirus G (EVG), and PSV as previously described [2]. Only PSV positive fecal samples were processed for virus isolation. In brief, fecal specimens were diluted in phosphate-buffered saline (PBS) to prepare a 10% suspension. The suspension was clarified by centrifugation and filtered through 0.22 µm filter (Merck Millipore, Burlington, MA, USA). One mL of filtered fecal supernatant was diluted 1:1 in DMEM containing 1% penicillin and streptomycin and 0% FBS and inoculated onto confluent PK15 cells, and incubated at 37 °C and 5% CO_2_ for 1 h. The inoculum was discarded and cells were washed two times with PBS and replaced with DMEM without FBS and incubated at 37 °C and 5% CO_2_ and observed daily for cytopathic effects (CPEs). Four days post-infection (dpi), cells were lysed by three freeze-thaw cycles and reinoculated into PK15 cells for three passages; at the same time, cell lysates were analyzed by RT-PCR for viral identification after every passage.

### 2.4. Viral Plaque Assay

To purify the isolated viruses, a plaque assay was performed as previously described with slight modifications [26]. PK15 cells were inoculated with a 10-fold serially diluted virus, and cells were then overlayed with 1% SeaPlaque agarose in DMEM containing 1% penicillin and streptomycin and 2% FBS. After plaque development, uniform and clear plaques were picked and reinoculated into the cell monolayer to harvest the positive clones. After three rounds of plaque purification, the virus clones were successfully obtained. The plates were also fixed with paraformaldehyde and stained with crystal violet for the observation of viral plaques.

### 2.5. Transmission Electron Microscopy (TEM)

The plaque-purified viruses were clarified by centrifuging at 10,000 rpm to remove cell debris, passed through 0.22 µm filter and centrifuged at 35,000 rpm for 4 h in a SW32Ti rotor (Beckman, Indianapolis, IN, USA). The resulting pellet was resuspended in DMEM and centrifuged through a 13 mL 20–50% (*w*/*v*) sucrose cushion (Beckman SW55Ti rotor, 4 h, 35,000 rpm, 4 °C). The virus particles that formed a white opalescent band at the interface of the sucrose solutions were collected, resuspended in DMEM, and centrifuged for 3 h (Beckman TLA55 rotor, 35,000 rpm, 4 °C). The purified virus was observed under TEM (Model H-7650, Hitachi, Tokyo, Japan). To further confirm the viral particles, cell supernatants were tested by RT-PCR with the specific primers as previously reported [2].

### 2.6. Virus Growth Kinetic

PK15 cells were infected in triplicate with PSV at a multiplicity of infection (MOI) of 0.01 to perform a growth curve of isolated PSVs. Cell supernatants were harvested at 1 h, 6 h, 12 h, 24 h, 36 h, 48 h, 60 h, and 72 h post-inoculation. Virus titers were measured by 10-fold serial dilutions in PK15 cells seeded into 96-well plates and calculated as 50% tissue culture infectious dose (TCID_50_) per mL according to the Reed-Muench method [27].

### 2.7. Whole-Genome Sequencing & Phylogenetic Analysis

Total RNA was extracted from plaque-purified PSV clones using the TIANamp virus RNA Kit (Tiangen Biotech, Beijing, China); cDNA was transcribed using the PrimeScript Double Strand cDNA Synthesis Kit (Takara, Dalian, China) and cDNA libraries were prepared using the Agencourt AMPure XP-Medium kit (Beckman Coulter, A63881, Brea, CA, USA) according to the manufacturer’s instructions. The prepared libraries were then sequenced using BGISEQ-500 Sequencing System (BGI, Shenzhen, China). After checking the quality of raw reads, based on the reference genome of *Sus scrofa* (Sscrofa11.1(GCA_000003025.6)), high-quality reads were processed to eliminate host contamination reads using BWA [28]. The non-host readings were mapped to the virus database using SOAP, and the candidate viruses were screened. Following the removal of low-quality readings, the remaining reads were de novo assembled using IDBA [29], SPAdes [30], and Edena [31]. Following the acquisition of contigs, local BLAST was used to identify viral species [32].

To understand the molecular characteristics of virus isolates, phylogenetic analyses of whole-genome and VP1 sequences were performed by comparing nucleotide sequences of isolated PSV strains with published PSV sequences in the GenBank database. Furthermore, alignments of amino acid sequences of the VP1 protein were also carried out. Nucleotide and amino acid sequences were aligned using Clustal W software. Phylogenetic trees were constructed via the maximum-likelihood method using MEGA v.6.0 software with the neighbor-joining method with Kimura 2-parameter and 1000 bootstrap replicates [33,34].

### 2.8. Generation of mAbs

The VP1 gene of PSV was amplified by RT-PCR and inserted into the prokaryotic expression vector pMAL-c5X. Recombinant VP1 was expressed as a maltose-binding protein-tagged (MBP)-tagged fusion protein in *Escherichia coli* ER2523 by adding 1 mM isopropyl-D-1-thiogalactoside, and examined by SDS-PAGE and Western blot. The recombinant-VP1 protein was purified using a pre-packed MBP Trap column under the AKTA liquid chromatography system (Cytiva, Marlborough, MA, USA), and evaluated by SDS-PAGE and Western blot. The purified fusion protein was used to immunize mice for mAb production. The mouse experiments were performed under the guidelines of the Animal Ethics Committee of Harbin Veterinary Research Institute of the Chinese Academy of Agricultural Sciences (approval number 210602-01). Method of mAb production used hybridoma technology according to the standard protocol described previously [35]. The mAb isotyping was performed using a mouse mAb isotyping kit (ThermoFisher Scientific, Waltham, MA, USA) according to the manufacturer’s instructions.

### 2.9. Western Blot Assay

The Western blot assay was carried out as previously described [36]. In brief, the lysates of PSV-infected PK15 cells and MBP-tagged recombinant VP1 protein were separated on SDS-PAGE gel and transferred to PVDF membranes. Membranes were incubated with either an mAb against VP1 protein (1:200), an anti-MBP mAb (1:10,000), or swine serum (1:100). Secondary antibodies were used with the IRDye 680 conjugated goat anti-mouse IgG (Li-Cor Biosciences, Lincoln, NE, USA) or goat anti-pig IgG (Biodragon-immunotech, Beijing, China) at a dilution of 1:10,000. Finally, membranes were scanned by an Odyssey infrared imaging system (Li-Cor Biosciences).

### 2.10. Immunofluorescence Assay

The indirect immunofluorescence assay (IFA) was conducted as previously described with slight modification [36]. Cell monolayers were inoculated with PSV at an MOI of 0.01 and incubated for 12 h. Cells were fixed with 4% paraformaldehyde, permeabilized with 0.1% Triton X-100, and blocked with 2% bovine serum albumin. A mAb specific for PSV-VP1 diluted 1:200 or swine serum at a 1:100 dilution was used as the primary antibody. The cells were then incubated with FITC-conjugated goat anti-mouse IgG 1:500 dilution (Thermo Fisher Scientific, Waltham, MA USA) or rabbit anti-pig IgG 1:50 dilution (Sigma, St. Louis, MO, USA). Nuclei were stained with 10 µg/mL DAPI (Solarbio, Beijing, China), and cells were then visualized under an inverted fluorescence microscope (Evos FL, Thermo-Fisher, Bothell, WA, USA).

### 2.11. Development of Indirect ELISA

The VP1-based indirect ELISA (iELISA) was developed based on previously described method with some modifications [37]. IFA and Western blot assays were used as a standard evaluating method for verifying positive and negative serum samples for iELISA optimization. Antigen concentration, serum and secondary antibody dilutions were optimized using checkerboard titration. Briefly, 96-well-plates were coated with purified VP1 protein (0.1–0.5 mg/mL) and incubated at 4 °C overnight. The plates were blocked with 200 uL of 5% skimmed milk for 2 h at 37 °C. After washing, 50 μL/well of PSV positive and negative serum samples (diluted 1:100 to 1:6400) were added and incubated for 2 h at 37 °C. After washing, 50 μL/well of HRP-conjugated goat anti-pig IgG (Sigma) (diluted 1:10,000 to 1:50,000) was added and incubated at 37 °C for 1 h. After washing, 50 μL/well of substrate was added and incubated for 15 min. Lastly, the reaction was stopped by adding 50 μL/well of 2 M sulphuric acid. The results were read at 450 nm optical density (OD_450_). The dilution with the highest OD_450_ ratio between positive and negative serum (P/N value) was considered optimal.

The cut-off value of iELISA was determined by analyzing 75 PSV-seronegative samples. To validate the specificity of iELISA, positive sera against other pathogens, including PEDV, porcine circovirus type 2 (PCV2), porcine reproductive and respiratory syndrome virus (PRRSV), classical swine fever virus (CSFV), African swine fever virus (ASFV), and Seneca Valley virus (SVV), were tested in triplicates and the mean OD_450 nm_ value. These sera were certified by Harbin Guosheng Biological Testing Technology Co., Ltd. (Harbin, China). To evaluate the validity of the iELISA, 92 swine sera were tested by the iELISA and IFA, respectively. The sensitivity was determined by the ratio of iELISA positive to IFA positive samples, whereas specificity was determined by the ratio of iELISA negative to IFA negative samples.

### 2.12. Epidemiological Investigation of PSV

To evaluate the frequency of PSV infection, a total of 516 swine serum samples were collected and tested using the iELISA developed here. For molecular detection a total of 291 porcine samples including feces, faecal swabs and intestinal tissues collected from 179 non diarrheic and 112 diarrheic pigs were investigated by RT-PCR with the specific primers as previously reported [2].

## 3. Results

### 3.1. Isolation of PSV

**Figure 1:**
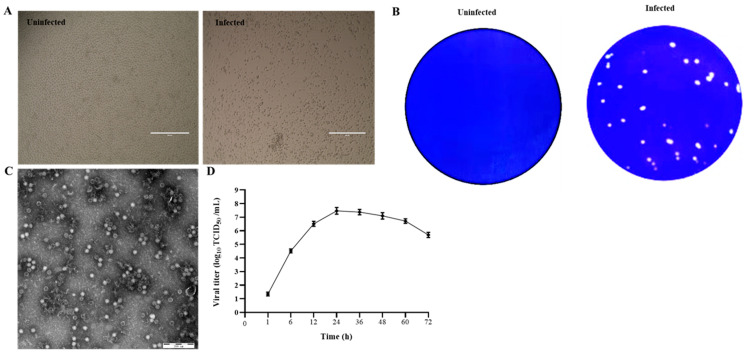
Isolation and identification of PSV. (**A**) Cytopathic effects in PSV-infected PK15 cells at 24 hpi. (**B**) Production of plaques of the PSV isolate in PK15 cells. (**C**) Picornavirus-like particles under TEM. (**D**) Growth kinetic of PSV.

PK15 cells were inoculated with six diarrheic stool samples that were positive to PSV but negative for other enteric viruses, including TGEV, PEDV, PDCoV, RVA, PSaV, PKV, and EVG. After 3 blind passages, cells inoculated with four samples started to show CPEs, characterized by cell rounding, shrinking, and detachment (Figure 1A). After three rounds of plaque purification, the purified virus developed uniform and clear plaques (Figure 1B). After ultracentrifugation, the plaque-purified viruses were examined under TEM, and the results showed that spherical, non-enveloped virus particles of approximately 32 nm in diameter were observed (Figure 1C). The RT-PCR and sequencing results showed that the isolated viruses were PSV. The virus growth curve results showed that the virus replicated fast and reached a peak of 10^7.8^ TCID_50_/mL at 24 h, suggesting that the cycle of PSV multiplication is completed within 24 h (Figure 1D).

### 3.2. Whole-Genome Sequence Analysis

Using next-generation sequencing technology, four entire genome sequences of PSV were obtained, named as PSV-12, PSV-14, PSV-15, and PSV-41 (GenBank access No. OM037653-OM37656). The results revealed that the entire genome of isolated PSVs ranged from 7569–7572 nucleotides, including 490 nucleotides of 5′-UTR sequence and 83 nucleotides of 3′-UTR sequence. By comparing the genomes of previously identified PSV strains with these four PSV isolates, the full ORF and cleavage sites of PSV were predicted. The length of the ORF of PSV-12, PSV-14, and PSV-15 strains was 6996 nucleotides, encoding 2332 amino acids, whereas, PSV-41 has longer ORF of 6999 nucleotides that encode 2333 amino acids due to a single amino acid insertion at position (896 P) at the 3′-end of ORF (Table S1).

The nucleotide and amino acid sequence identities among four PSV isolates ranged from 92–99.9% and 98.6–100%, respectively. While PSV-12, PSV-14, and PSV-15 were isolated from the same farm, their nucleotide (99.7–99.8%) and amino acid sequences (99.9–100%) similarities suggest that similar PSV strains were circulating on the farm. In contrast, PSV-41, isolated from another farm, showed a relatively low identity in nucleotide (91.1–91.2%) and amino acid sequences (98.6–98.7%) with the other three PSVs. These data indicate that PSVs circulating on two farms are genetically distinct. Furthermore, identity comparisons of whole genome among PSVs demonstrated that PSVs identified in this study had 77.7–92% and 84.3–98.9% similarities in nucleotide and amino acid sequences, respectively, compared to the 66 PSV reference strains available in the GenBank database (Table S2).

**Figure 2:**
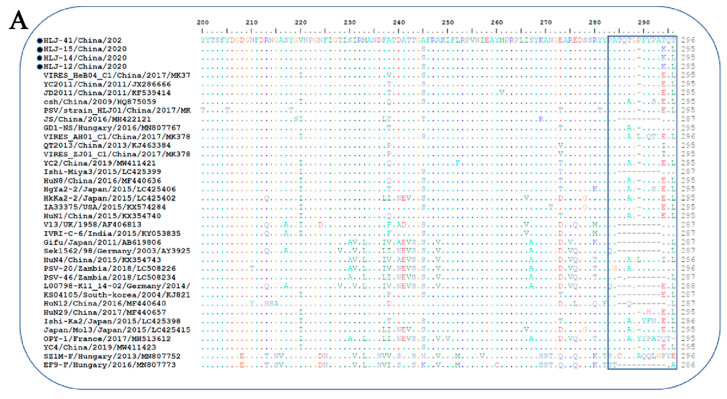

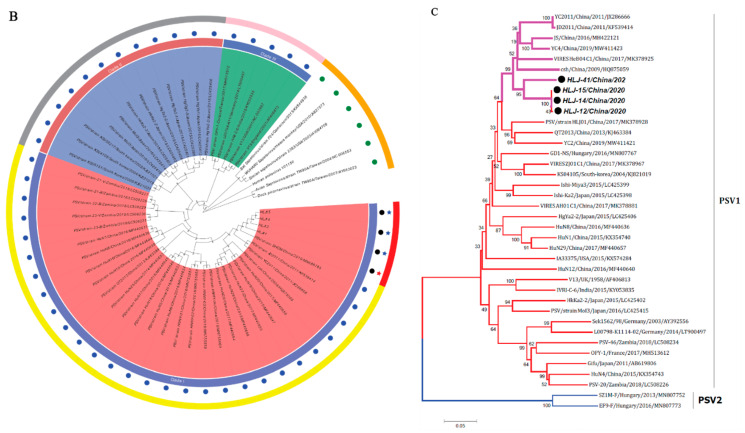
Sequence analysis of the isolated PSV strains. (**A**) Alignment of 3′-partial amino acid sequences of VP1 of PSV strains. Sequences in boxes showed the hypervariable region in the C-terminus of VP1. The strains identified in the present study were indicated by black dots; (-), missing amino acids. (**B**,**C**) Phylogenetic analysis of PSV based on the complete nucleotide sequence and VP1 gene respectively. The trees were generated by using MEGA v.6.0 with the neighbor-joining method with the Kimura 2-parameter with 1000 bootstrap replication. The black circle indicates PSV isolated in this study. The scale bars represent the number of substitutions per site.

The VP1 gene of three PSV strains (12, 14, and 15) contained 882 nucleotides and encoded 294 (aa) protein, according to an alignment analysis of capsid VP1 of PSVs identified in this study with those of other known PSVs. The PSV-41 strain, on the other hand, had three more nucleotides (885), one extra proline inserted between aa 286 and 287 of the VP1 (Figure 2A). Homology comparison of the VP1 gene of isolated PSVs with reference strains revealed sequence similarity ranging from 62.6% to 90.6% nucleotide and 60.6% to 99% amino acid (Table S2). Moreover, the isolated PSV strains had a sequence similarity of 90.7–100% nucleotide and 96.4–100% amino acid with each other.

### 3.3. Phylogenetic Analysis

The phylogenetic tree based on complete ORF nucleotide sequences was constructed using the current PSVs sequences and representative PSV sequences from the NCBI database (Table S2). As shown in Figure 2B, PSVs were phylogenetically classified into three clades (clade I—clade III), correlated with their geographical location. Clade I included Chinese and Zambian PSV strains. Clade II consisted of PSV strains detected in South Korea and Japan, while clade III was composed of the strains identified in French, Germany, India, USA, and UK. The four PSV strains isolated in this study were grouped into clade I and formed a monophyletic clade with Chinese and Zambian strains.

The phylogenetic tree based on VP1 protein revealed that all PSVs were separated into two clades, each corresponding to two genotypes, named PSV-1 and PSV-2 (Figure 2C). The PSV strains isolated here were classified in the typical PSV-1 clade, along with previously described PSV strains; whereas two Hungarian strains, SZ1M-F and EF9-F, were classified in the PSV-2 clade. The four PSV isolates have formed a monophyletic clade, with topological structure and branching of the evolutionary tree in the same manner of complete ORFs.

### 3.4. Expression and Purification of VP1 Protein

**Figure 3:**
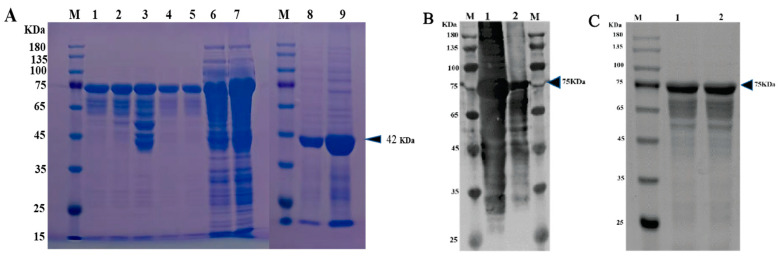
Expression and purification of PSV-VP1 protein in *E. coli*. (**A**) SDS-PAGE analysis of the MBP-tagged recombinant VP1-protein expression (~75 KDa). Lane M, protein marker; Lanes 1–5, purified bacterial cell lysates of MBP-tagged VP1 at different concentrations; Lines 6 and 7, unpurified bacterial cell lysate of MBP-tagged VP1; Line 8, sediment of bacterial cell lysate of empty vector, Line 9, supernatant of bacterial cell lysate of =empty vector (~42 KDa). (**B**) Western blot analysis of unpurified recombinant VP1-protein using anti-MBP tag antibody. Lane 1, 0.2 mg/mL of unpurified VP1 protein; Lane 2, 0.1 mg/mL of unpurified VP1 protein. (**C**) Western blot analysis of purified VP1-recombinant protein using anti-MBP tag antibody. Lane 1, 0.1 mg/mL of purified VP1 protein; Lane 2, 0.2 mg/mL of purified VP1 protein.

The VP1 protein of the PSV-14 was expressed as an MBP-tagged fusion protein as shown in the SDS-PAGE result (Figure 3A). The expression of recombinant VP1 protein of approximately 77 kDa was confirmed by Western blot using an MBP-specific antibody (Figure 3B). The recombinant VP1 protein was purified by the AKTA liquid affinity chromatography system using an MBP trap column. The purified recombinant VP1 protein was evaluated by Western blot, and the results showed that the recombinant VP1 protein was efficiently purified (Figure 3C).

### 3.5. Production and Application of mAbs

**Figure 4:**
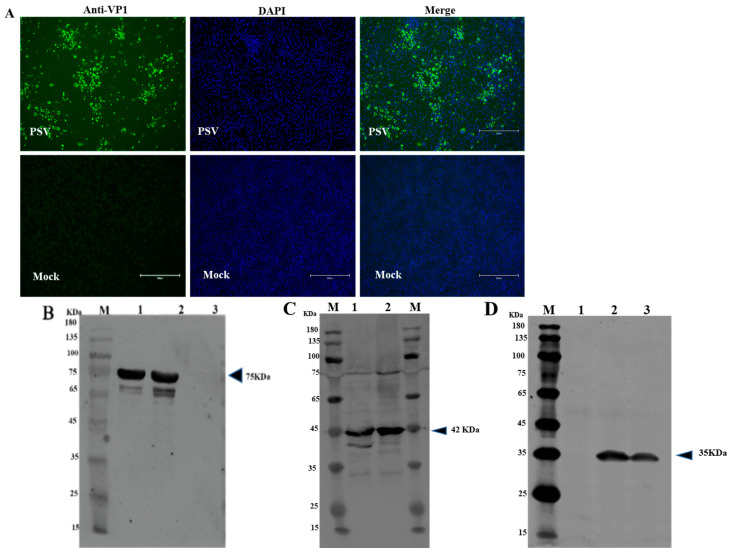
Identification of mAb against VP1 of PSV. (**A**) Identification of mAb against PSV-VP1 by IFA. PK15 cells infected with PSV at MOI of 0.01. IFA was performed using generated mAb against VP1. (**B**) Western blot analysis of the purified recombinant protein from *E. Coli*. Lane M, protein marker; Lane 1, 0.1 mg/mL purified MBP-tagged VP1-recombinant protein; Lane 2, 0.2 mg/mL MBP-tagged VP1-recombinant protein; Lane 3, purified MBP. (**C**) Western blot analysis of the expressed MBP in empty vector. Lane M, protein marker; Lane 1, sediment of bacterial cell lysate, Line 2, supernatant of bacterial cell lysate. (**D**) Western blot analysis of PK15 cells infected with PSV. Lane M, protein marker; Lane 1, mock cells; Lane 2, PK15 cells infected with PSV; Lane 3, ST cells infected with PSV.

The mouse with the highest antibody titer against recombinant VP1 was sacrificed, and splenocytes were fused with SP2/0 myeloma cells to create a confluent hybridoma that was screened using the purified recombinant VP1 protein as coating antigen. Positive colonies were selected and sub-cloned to yield one hybrid cell per well. Five positive clones were eventually generated and classified as 1C3, 1F6, 2D2, 3D11, and 4G1. Since all the five mAbs have similar characteristics (belonged to isotype IgG2a with kappa light chain), we randomly selected and evaluated 4G1. The specificity of 4G1 was then evaluated by IFA and Western blot assays. IFA results showed that green fluorescence was observed in PSV-infected PK15 cells and there was no fluorescence in mock infected-PK15 cells (Figure 4A). Western blot results revealed that mAb 4G1 was able to recognize a 77 kDa of MBP-tagged VP1 protein (Figure 4B) and a ~35 kDa VP1 protein in PK15 cells infected with PSV (Figure 4D). The cell lysate transformed with empty plasmid was set up as a positive control for MBP expression (~42 KDa) (Figure 4C).

### 3.6. Cell Susceptibility Test to the PSV Isolate

**Figure 5:**
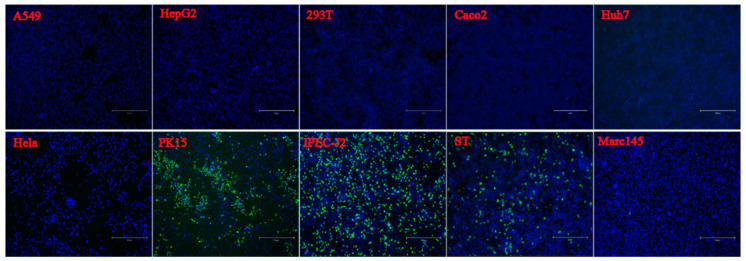

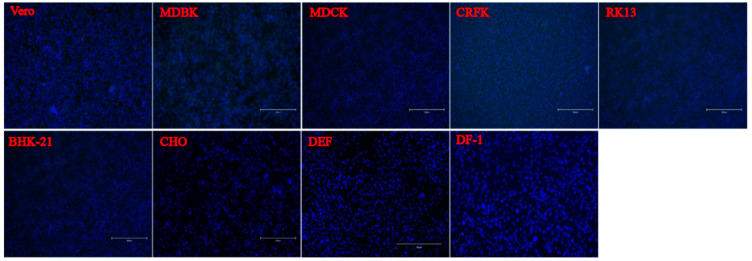
Susceptibility of PSV to cell lines derived from various species. Cell lines of Human (A549, HeLa, Huh-7, HepG2, Coca2 and 293T), Swine (PK15, IPEC-J2, and ST), Monkeys (Marc-145 and Vero), Bovine (MDBK), Canine (MDCK), Feline (CRFK), Rabbit (RK13), Hamster (BHK-21 and CHO), Duck (DEF) and Chicken (DF-1) were infected with PSV at an MOI of 0.01, and fixed at 24 hpi. Cell monolayers were determined by IFA using PSV mAb.

To evaluate the infection and replication ability of the isolated PSV strain, cell line susceptibility test was performed using nineteen cell lines derived from various species (Table 2). The results revealed that the isolated PSV strain could only replicate in cell lines from swine origin and showed CPEs characterized by cell rounding, shrinking, and detachment at 24 h after infection, while other cell lines did not show CPEs. The evidence of productive infection of PSV in the swine cell lines was confirmed by the detection of viral VP1 protein expression using IFA with mAb 4G1 (Figure 5), suggesting that additional porcine primary factors might be necessary for PSV infection and replication.

### 3.7. Establishment and Optimization of iELISA

**Figure 6:**
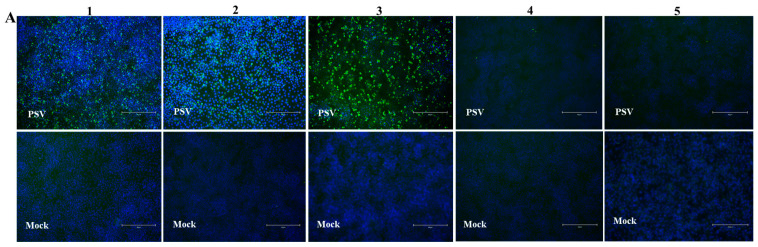

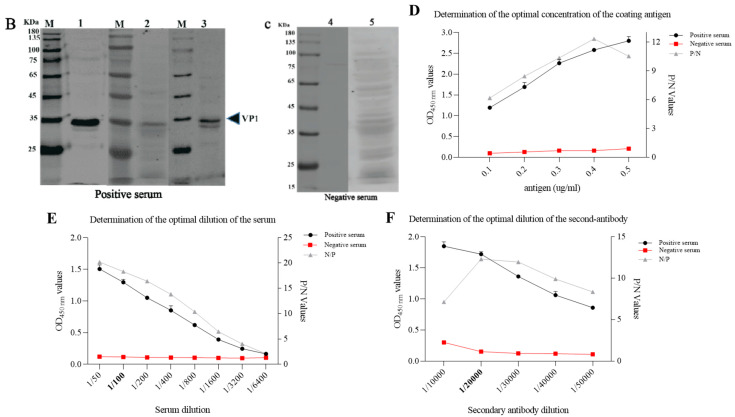
Optimization of reaction conditions of iELISA. (**A**) Verification of PSV-negative and positive swine sera by IFA. PK15 cells were inoculated with PSV at MOI of 0.01. IFA was performed using swine positive serum # 1, 2, and 3, and negative serum # 4 and 5. These are the representative sera of all the tested sera. (**B**,**C**) Confirmation of PSV positive and negative serum by Western blot. Protein lysates of PK15 cells infected with PSV were detected with PSV positive and negative swine serum respectively. (**D**–**F**) Optimization of the concentrations of coating antigen, serum dilutions and second antibody respectively.

IFA was used as a standard evaluating method for verifying seropositive and seronegative samples for iELISA optimization. In total 169 serum samples (72 positive and 97 negative samples), were collected from positive and negative swine by RT-PCR. The appropriate serum dilution for the IFA was determined by serial dilution (from 1:20 to 1:200) and found that the optimal dilution of serum was 1:100. The *iELISA* positive and negative sera were then confirmed by IFA, see the representative data in Figure 6A. To validate the serum samples, Western blot assay was further performed in PSV-infected PK15 cells by testing 48 samples (28 positive and 20 negative samples) randomly selected from the sera tested by IFA. We found that Western blot results were consistent with IFA, as shown in the representative data in Figure 6B,C. Interestingly, the VP1 protein with a molecular weight of 35 kDa was detected by the PSV-positive sera, but was not detected by the negative serum. Though PSV has four structural proteins, including VP1, VP2, VP3 and VP4, only VP1 was found to react with PSV-positive sera, implying that VP1 is the most abundant and strongest antigenic protein in the PSV particle.

To optimize the iELISA, the checkerboard titration was performed to determine the optimal concentration of the antigen and the serum. The results showed that the optimal antigen coating concentration was 0.2 µg/mL and the optimal dilution for serum was 1:100 (Figure 6D,E). Using this optimal dilution of coating protein and primary antibody, the optimal dilution of the HRP-conjugated goat anti-porcine IgG was determined to be 1:20,000, as shown in Figure 6F.

### 3.8. Determination of the Cut-Off Point and Evaluation of iELISA Specificity and Sensitivity

**Figure 7:**
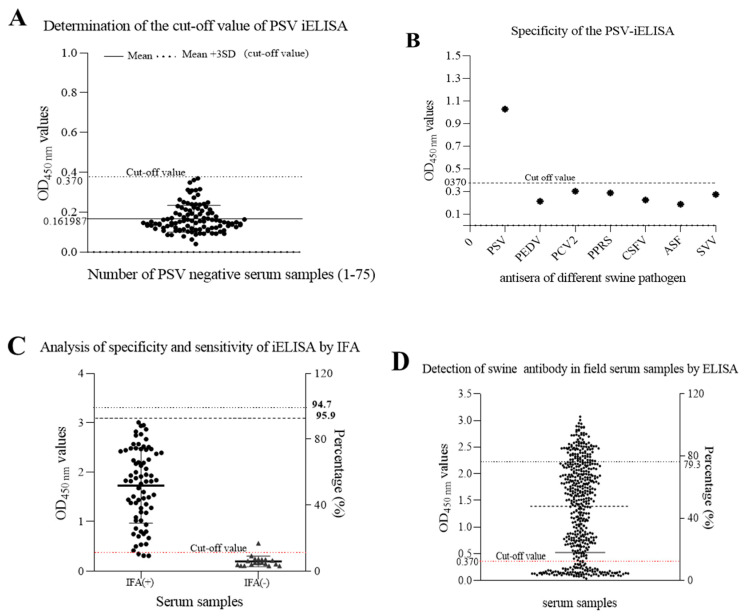
Detection of anti-PSV antibodies in swine serum by the developed iELISA. (**A**) seventy-five PSV-negative serum samples were tested using iELISA and the mean OD_450_ value of PSV-negative serum plus three standard deviations (SDs) were used to calculate the cutoff value. (**B**) Identification of iELISA specificity by cross-reaction test. Positive swine serum against PSV, PEDV, PCV2, PRRS, CSFV, ASFV and SVV were tested by iELISA. The average of OD_450_ values were calculated to determine the tested serum according to the cut-off value. (**C**) The sensitivity and specificity of the developed ELISA were assessed based on the IFA result. (**D**) Detection of anti-PSV antibodies from field swine serum samples by the developed iELISA.

Seventy-five PSV-negative serum samples that were collected from swine negative to PSV by RT-PCR and confirmed by IFA and validated by Western blot assay, were used to determine the cut-off point. The cut-off point was specific to each assay and was based on the OD450nm of the 75 negative samples. Based on the mean OD_450 nm_ plus 3 SD (0.161987 + 30.069591; n = 75; Max = 0.361; Min = 0.063), the cutoff value was determined to be 0.370 (Figure 7A). The negative-positive threshold was consequently set at 0.370, and serum samples with an absorbance of greater than 0.370 at OD450nm were considered PSV seropositive, and vice versa.

To determine the specificity of this iELISA, porcine positive sera against other viruses, including PEDV, PCV2, PRRSV, CSFV, ASFV, and SVV, were examined. The results showed that there was no cross-reactivity of the newly developed iELISA with other porcine viruses, as shown in Figure 7B, and the average OD_450_ values of positive serum for PEDV, PCV2, PRRS, CSFV, ASFV, and SVV, were 0.213, 0.287, 0.302, 0.224, 0.187, and 0.273, respectively, indicating that the established iELISA was specific for the detection of PSV antibodies.

Furthermore, a total of 92 porcine sera collected from the field were tested in parallel using the iELISA and the IFA test. The results showed that positive and negative serum numbered 70 and 18 by IFA, but 73 and 19 by iELISA. In total, 70 samples were positive and 18 negatives in both cases, which represents a concurrence for 95.7%. Three positive samples in the iELISA test were negative in the IFA test, and one sample which was negative in the iELISA test have become positive in the IFA test. Hence, the sensitivity of iELISA was 95.9% among PSV-seropositive samples, and the specificity was 94.7% among PSV-seronegative samples using IFA as standard evaluation method (Figure 7C, Table 3).

### 3.9. Epidemiological Investigation of PSV

To assess the frequency of PSV infection, a total of 516 swine serum randomly collected from pigs with different ages were investigated by the developed iELISA. As shown in Figure 7D, the positive rate was 79.3% (409/516), indicating that PSV infection prevalent in swine in China. Furthermore, molecular detection of PSV in a total of 291 porcine samples, including feces, faecal swabs and intestinal contents collected from 179 non diarrheic and 112 diarrheic pigs were investigated by RT-PCR. As shown in Table 4, out of 291 samples, 44% (128/291) samples were positive for PSV; of these PSV positive pigs, 54.5% (61/112) were diarrheic and 37.43% (67/179) were non diarrheic. PSV was identified in 28.4% (27/95) of the suckling pigs, 62.1% (64/103) of the nursery pigs and 39.8% (37/83) of the fattening pigs. The high PSV prevalence was found in nursery and fattening pigs. The data revealed that the prevalence rate of PSV was significantly higher in diarrheic animals than in non-diarrheic animals.

## 4. Discussion

PSV has been implicated in various swine diseases and associated with significant economic losses in the swine industry. Due to subclinical manifestations and coinfections with other pathogens, PSV infection frequently goes unnoticed or overlooked [24,38]. Thus, when mass diarrhea occurs in a swine herd, most researchers focus on other enteric viruses, while PSV, which can cause similar symptoms, receives less attention [39,40], as they do not believe PSV is consistently pathogenic. However, a previous report demonstrated that PSV infection is more prevalent in symptomatic animals than asymptomatic [41]. In this study, we isolated and genetically characterized four PSV strains (PSV-12, PSV-14, PSV-15, and PSV-41) from diarrheic pigs. The isolated viruses were confirmed by RT-PCR, sequencing, TEM, and IFA assays. Since these four PSV isolates have relatively similar characteristics, the following experiments were partially carried out with PSV-14 strain.

To evaluate viral tropism, cell susceptibility was tested on 19 cell lines derived from various host species. According to previous reports from Japan [6] and China [1], PSV infection is susceptible to hepatocarcinoma cell lines (PLC/PRF/5 and HepG2/C3a), 293T, Vero E6, primary green monkey kidney cells (PGMKC), and BHK-21. Contrary to this, our findings indicated that isolated PSV strains were able to replicate only in porcine cell lines. This is comparable to the findings of Kim, Kang [8], suggesting that porcine primary factors might be necessary for viral replication.

To provide detailed analysis of genome characterization, complete genomes of isolated PSVs were sequenced and phylogenetic relationships among PSV strains were determined. The alignments of whole-genome sequences demonstrated that the nucleotide and amino acid sequence identities of PSVs isolated in the current study ranged from 92–99.9% and 98.6–100%, respectively, and shared 77.7–92% and 84.3–98.9% similarities in nucleotide and amino acid level, respectively, with PSV reference strains available in GenBank. The polyprotein genes phylogenetic analysis revealed that the PSV strains isolated in this study are more closely related to PSVs previously detected in China than PSVs isolated from other countries and classified into distinct clusters based on geographic locations [1,19,22]. These findings indicate that the isolated PSVs share the closest genetic relationship with the strains isolated in China, implying that the PSV strains prevalent in China most likely evolved from common ancestors.

Currently, many picornaviruses are solely classified based on VP1 sequence identities [3]. phylogenetic analysis of a partial VP1 gene sequence has been used for classification of PSV isolates [21]. Due to the absence of classification criteria, PSV have long been contained only one genotype PSV-1 [42]. In contrast, novel PSV strains (SZ1M-F and EF9-F) were recently described in Hungary. They were thought to represent a novel genotype of PSV and were designated as PSV-2 [14]. The VP1 sequences of 34 reference PSVs from GenBank were phylogenetically analyzed, and the results indicated that the PSVs isolated in the current study belonged to the typical genotype (PSV-1) and clustered together, similar to the tree topology of polyprotein gene sequences. Picornavirus capsids are made of four structural proteins (VP1–4), and the outer surface structure of picornaviruses is largely determined by the spatial folding and mutual extension of VP1, VP2, and VP3 [43]. The C-terminus of VP1–3 is situated on the viral outer surface, while the N-terminus is located on the inner surface. Thus, amino acid changes in the C-terminus of VP1–3 have been proposed to affect picornavirus antigenicity [44]. In the present study, one amino acid insertion near the C-terminus of VP1 was found in PSV-41. This insertion is also present in other strains isolated in China, Japan, and Zambia. Possibly, changes in the C terminus of the PSV-VP1 may be related with escape from the host immune response. However, more research is needed to determine the role of those mutations as well as their distinct biological properties.

Despite the importance of exploring the virus epidemiology, specific antibody-based reagents and a serological assay that could be used to detect PSV infection are not available yet. Currently, diagnosis of PSV is mainly based on the detection of nucleic acid by PCR and virus isolation [1,15,19,27]. However, detection of virus antigens and antibodies in diagnostic specimens may be more accurate and less expensive, enabling faster and broader field use and confirming the presence of virus in cell culture [45]. In contrast, identification of the most antigenic viral proteins has a vital role in improving serological diagnostic assays. The PSV capsid protein VP1 is the most immunodominant protein, containing several major antigenic epitopes [3,5]. Based on these facts, the purified recombinant VP1 protein was used as immunogen to produce mAb and as coating protein to establish iELISA. The PSV specific mAb generated in this study, providing sensitive identification of PSV, are well adapted for immunofluorescence and western blot assays to detect PSV antigens. In addition, could be of substantial value in detection of PSV antigens in a variety of applications such as early confirmation of virus isolation and virus titrations and detection of virus in clinical tissues.

The iELISA for anti-PSV antibody was successfully developed and its accuracy was optimized. The results revealed that the developed iELISA has a sensitivity of 95.9% and a specificity of 94.7%, respectively. Moreover, no cross-reactivity with antibodies against known enteric viruses was observed, indicating that the developed iELISA was specific, sensitive and could be used as a rapid and reliable diagnostic method. The accuracy of iELISA was further checked by testing 516 swine serum samples. Notably, the result revealed that a seropositive rate of PSV was 79.3% (409/516). Similar result by using IFA, a PSV seropositive rate of 80.23% (207/258) was reported in swine farms [1]. In addition, PCR based detection revealed 44% (128/291) of the investigated pigs were PSV positive. Together, these findings indicate that PSV is highly prevalent among swine in China. Although the serum and fecal samples were collected from the same farms, the iELISA based detection method showed a higher prevalence of PSV, compared to the PCR based detection. Since the fecal samples did not match up with the serum samples, we could not make a direct connection between virus detection rates and iELISA results. However, the high positive rate of iELISA may be due to a high likelihood of past viral exposure. Moreover, the low virus detection rates may be attributed to the virus shedding at early stage of infection. It will be interesting to further analysis the correlation of the virus distribution and sero-prevalence. These data indicate that the developed iELISA was more sensitive, enabling wider field-based application. Further, the overall prevalence of PSV was much higher in diarrheic animals than in non-diarrheic animals. These results were consistent with previous studies showing that PSV is widely prevalent in pigs with diarrhea [21,46]. Thus, when controlling swine diarrhea, PSV infection should not be overlooked.

## 5. Conclusions

In summary, this report has described the isolation and characterization of PSV strains from diarrheic piglets. The PSV mAbs described herein can be used to validate virus isolation results in cell culture, identify viral antigens in clinical specimens. In addition, for the first time, viral VP1 protein-based iELISA has been established to effectively detect PSV-specific antibodies from blood samples, would be a useful tool for epidemiological studies of PSV. Taken together, these results revealed that PSV is highly prevalent among swine in China. However, to accurately evaluate the impact of PSV infection, additional research is necessary to better understand the epidemiology of the virus and the molecular mechanisms driving its pathogenicity.

## Figures and Tables

**Table 1:** Fecal samples background.

Production Stage	Clinical Status		Total
	Diarrheic	Asymptomatic	
Suckling (<28 days)	29	66	95
Nursery (28–70 days)	45	58	103
Fattening (>70 days)	38	55	93
Total	112	179	291

**Table 2:** Summary of cell lines susceptibility to PSV as determined by CPE and IFA.

NO	Name	ATCC^®^ Number	Tissue Origin	Species	CPE	IFA
1	293T	CRL-11268	Embryonic kidney	human	−	−
2	A549	CCL-185EMT	Lung carcinoma	human	−	−
3	Hela	CCL-2	Cervix adenocarcinoma	human	−	−
4	HepG2	HB-8065	Hepatocellular carcinoma	human	−	−
5	Huh7	N/A	Hepatocellular carcinoma	human	−	−
6	Caco2	HTB-37	Colon adenocarcinoma	human	−	−
7	PK15	CCL-33	Porcine Kidney	swine	+	+
8	ST	CRL-1746	Swine testicular	swine	+	+
9	IPEC-J2	N/A	Small intestinal epithelium	swine	+	+
10	Marc145	N/A	African green monkey kidney	monkey	−	−
11	Vero E6	CRL-1586	African green monkey kidney	monkey	−	−
12	MDBK	CCL-22	Madin-darby bovine kidney	bovine	−	−
13	MDCK	CCL-34	Madin-darby canine kidney	canine	−	−
14	CRFK	CCL-94	Crandell Reese Feline Kidney	feline	−	−
15	RK13	CCL-37	Rabbit kidney	rabbit	−	−
16	BHK-21	CCL-10	Baby hamster kidney	hamster	−	−
17	CHO	CCL-61	Chinese hamster ovary	hamster	−	−
18	DEF	CCL-141	Duck embryo fibroblasts	duck	−	−
19	DF-1	CRL-12203	Embryo fibroblasts	chicken	−	−

(+) infection; (−) No infection.

**Table 3:** Comparison of the iELISA with the IFA results.

		IFA Results		
		Positive	Negative	Total
iELISA results	Positive	70	3	73
	Negative	1	18	19
	Total	71	21	92
Data analysis	Sensitivity	95.9%		
	Specificity		94.7%	
	Coincidence rate			95.7%

**Table 4:** Prevalence of PSV infection in diarrheic and asymptomatic animals.

	Clinical Status		Total
Production stage	Diarrheic (112)	Asymptomatic (179)	291
Suckling			
Positive	9(31.0%)	18(27.3%)	27(28.4%)
Negative	20(69.0%)	48(72.7%)	68(71.6%)
Nursery			
Positive	35(77.8%)	29(50.0%)	64(62.1%)
Negative	10(22.2%)	29(50.0%)	39(37.9%)
Fattening			
Positive	17(44.7%)	20(36.4)	37(39.8)
Negative	21(55.3%)	35(63.6)	56(60.2)
Total			
Positive	61(54.5%)	67(37.4%)	128(44%)
Negative	51(45.5%)	112(62.6%)	163(56%)

## Data Availability

Not applicable.

## Supplementary Materials

The following are available at https://www.mdpi.com/article/10.3390/v14020349/s1, Table S1: Complete genome sequence details of isolated PSV strains. Table S2: Homology comparison of whole genome and VP1 of isolated PSV with reference strains.
