# Supplementary material for: Isolation, Characterization, and Molecular Detection of Porcine Sapelovirus
Source: Viruses. 2022 Feb 8;14(2):349. doi: 10.3390/v14020349 (PMC8877214; doi:10.3390/v14020349)
Supplement: Supplementary file 1 [file viruses-14-00349-s001.zip › viruses-1565529-supplementary.pdf]

Table S1**.** Complete genome sequence details of isolated PSV strains.

Strain	Nucleotide and deduced amino acid lengths of encoded proteins region in isolated PSVs														
	5’ UTR	L	VP4	VP2	VP3	VP1	2A	2B	2C	3A	3B	3C	3D	ORF	3’ UTR	Total length
PSV-41	490[a]	252	159	714	702	885	678	315	996	300	66	546	1386	6999	83	7542
		(84[b])	(53)	(238)	(234)	(295)	(226)	(105)	(332)	(100)	(22)	(182)	(462)	(2333)		
PSV-15	490	252	159	714	702	882	678	315	996	300	66	546	1386	6996	83	7542
		(84)	(53)	(238)	(234)	(294)	(226)	(105)	(332)	(100)	(22)	(182)	(462)	(2332)		
PSV-14	490	252	159	714	702	882	678	315	996	300	66	546	1386	6996	83	7566
		(84)	(53)	(238)	(234)	(294)	(226)	(105)	(332)	(100)	(22)	(182)	(462)	(2332)		
PSV-12	490	252	159	714	702	882	678	315	996	300	66	546	1383	6996	83	7491
		(84)	(53)	(238)	(234)	(294)	(226)	(105)	(332)	(100)	(22)	(182)	(461)	(2332)		

Abbreviation: UTR, untranslated region; ORF, open reading frame; [a], nucleotide; [b], amino acid in brackets.

Table S2. Homology comparison of whole genome and VP1 of isolated PSV with reference strains

No	Strain	Year	Host	Country	Accession No.	Nucleotide (nt) and amino acid (aa) sequence identity (%)			
						WGS		VP1	
						nt	aa	nt	aa
1	V13	1958	Swine	UK	AF406813	85.6	92.7-93	78.2-78.5	83.5-85.2
2	V13	2001	Swine	USA	NC_003987	85.6	92.7-93	78.2-78.5	83.5-85.2
3	HgYa2-2	2015	Swine	Japan	LC425406	87.9-88.1	96-96.4	81.7-81.9	89.6-90.6
4	HgYa1	2016	Swine	Japan	LC425413	87.8-88	96-96.4	79.9-80.3	88.8-89.5
5	Japan/Mol3	2016	Swine	Japan	LC425415	88.2-88.3	96.2-96.6	81.2-81.4	88.2-89.6
6	Japan/Mol2	2016	Swine	Japan	LC425414	88.3-88.6	96.6-96.9	81.6—82.8	90.5-91.5
7	HgTa2-2	2015	Swine	Japan	LC425404	87.7-88	95.8-96.1	76.1-77	85.7-86.4
8	HkKa2-3	2015	Swine	Japan	LC425403	88-88.1	96.2-96.3	80.5-81.2	86.8-88.1
9	HkKa2-2	2015	Swine	Japan	LC425402	88-88.2	96.2-96.3	80.6-81.4	86.2-88.2
10	HgTa2-1	2015	Swine	Japan	LC425401	87.9-88	96-96.1	81.6-82.4	90.2-92.5
11	HgOg2-5	2015	Swine	Japan	LC425395	87.9-88.3	96-96.3	77.4-77.6	86.7-87.1
12	Ishi-Ka2/2015	2015	Swine	Japan	LC425398	86.6-86.8	95.3-95.5	81.3-82.8	89.9-90.9
13	Ishi-Miya3	2015	Swine	Japan	LC425399	86-86.7	94.8-94.9	80.7-81.1	89.9-90.9
14	L00798-K11_14-02	2014	Swine	Germany	LT900497	85.4-86.2	92.7-92.8	74.7-75.6	77.1-77.4
15	OPY-1	2017	Swine	France	MH513612	85.7-85.8	93	75.9-77	78.5-79.5
16	KS04105	2004	Swine	South Korea	KJ821019	88.1-88.3	96.7-96.8	82.4-83	92.6-94.3
17	KS05151	2005	Swine	South Korea	KJ821020	88.7-88.8	97.2-97.4	83.1-83.4	94.2-95.6
18	KS055217	2005	Swine	South Korea	KJ821021	87.5-87.8	95.4-95.6	79.3-80	87.4-88.4
19	IVRI-C-6	2015	Swine	India	KY053835	85.4-85.8	93.1-93.2	78.3-80.2	85.2-87.2
20	Gifu	2011	Swine	Japan	AB619806	-	-	74.8-75.3	79.1-80.1
21	Sek 1562/98	2003	Swine	Germany	AY392556	-	-	76.3-77.4	78.1-78.5
22	PSV-20	2018	Swine	Zambia	LC508226	87.4-87.7	94.8-95.2	76.5-76.6	78.8-79.5
23	PSV-21-V	2018	Swine	Zambia	LC508227	87.4-87.5	94.7-95	79.5-79.8	86.7-87.1
24	PSV-21-B	2018	Swine	Zambia	LC508228	87.4-87.6	94.7-95	79.5-79.8	86.7-87.1
25	PSV-22-B	2018	Swine	Zambia	LC508229	87.4-87.6	94.7-94.9	79.5-79.8	86.4-86.7
26	PSV-23-V	2018	Swine	Zambia	LC508230	87.3-87.6	94.6-94.7	79.4-79.6	85.7-86.4
27	PSV-23-B	2018	Swine	Zambia	LC508231	87.3-87.6	94.6-94.8	79.6-79.9	86.1-86.7
28	PSV-46	2018	Swine	Zambia	LC508234	87.1-87.3	94.1-94.2	74.4-75.8	79.8-80.1
29	YC2011	2011	Swine	China	JX286666	91.2	98.2-98.4	87.8-90.6	96.3-99
30	JD2011	2011	Swine	China	KF539414	90.8-90.9	98.1-98.2	87.6-90.4	95.6-98.3
31	csh	2009	Swine	China	HQ875059	90.4-90.5	97.4-97.6	87.1-87-9	93.6-95.3
32	JS/CHN/2016	2016	Swine	China	MH422121	-	-	84.5-87.2	92.3-93.9
33	VIRES_HLJ01_C1	2017	Swine	China	MK378928	89.2-89.4	85.1-85.2	85.9-87.3	93.9-95.6
34	GD1-NS/PSV/HUN/2016	2016	Swine	Hungary	MN807767	-	-	85.6-85.7	93.3-93.9
35	VIRES_AH01_C1	2017	Swine	China	MK378881	89.4-89.8	95.2-95.3	83.7-84.7	91.6-92.9
36	QT2013	2013	Swine	China	KJ463384	90.1-90.4	97.8-98.2	84.5-84.8	93.6-94.3
37	IA33375/2015	2015	Swine	USA	KX574284	86.4-86.8	95.7-95.8	81.9-82.3	91.9-92.9

38	HuN1	2015	Swine	China	KX354740	88.3-88.4	96.5-96.7	81-83.5	90.6-92.3
39	HuN4	2015	Swine	China	KX354743	86.8-87	94.9-95.2	75.6-76.4	79.5-79.8
40	VIRES_ZJ01_C1	2017	Swine	China	MK378967	89.2-89.6	95-95.3	84.1-84.4	93.9-95.3
41	VIRES_HeB04_C1	2017	Swine	China	MK378925	90.5-90.7	98.7-98.9	88.7-90.1	96-98.7
42	SHCM	2019	Swine	China	MN685785	90.2-91.2	98.1-98.3	83.7-85.5	94.6-94.9
43	YC4	2019	Swine	China	MW411423	-	-	86.5-89	95.3-97.6
44	YC2	2019	Swine	China	MW411421	-	-	83.8-83.9	92.3-93.6
45	HNNY-04	2018	Swine	China	MN755860	89-89.5	93.9-94	80.8-81.2	85.4-86.8
46	HNNY-03	2018	Swine	China	MN755859	89.1-89.5	94-94.1	80.9-81.3	85.4-86.8
47	HNNY-02	2018	Swine	China	MN755858	90.5-91	95.9-96	83.7-85	91.8-93.9
48	HNNY-01	2018	Swine	China	MN755857	90.5-90.9	95.9-96.1	83.1-84.3	91.2-93.2
49	HuN5	2016	Swine	China	MF440633	91-91.4	98.2-98.4	85.2-87.3	92.9-94.9
50	HuN6	2016	Swine	China	MF440634	91-91.5	98.4-98.6	85.1-88.2	95.2-96.9
51	HuN7	2016	Swine	China	MF440635	89.5-89.7	97.4-98.6	82.2-83.4	90.5-91.5
52	HuN8	2016	Swine	China	MF440636	89.7-89.9	97.5-97.6	81.6-83.4	90.6-91.9
53	HuN10	2016	Swine	China	MF440638	89.8-90.2	97.2-97.4	81.4-83.7	90.8-92.2
54	HuN12	2016	Swine	China	MF440640	86.8-87.1	95.2-95.4	80-80.5	87.2-87.5
55	HuN16	2016	Swine	China	MF440644	89.5-89.7	97.1	82-83.4	90.5-92.2
56	HuN17	2016	Swine	China	MF440645	91.3-91.9	98.4-98.5	84.8-87.6	94.6-96.3
57	HuN18	2016	Swine	China	MF440646	91.4-92	98.4-98.5	84.7-87.7	94.6-96.3
58	HuN19	2017	Swine	China	MF440647	91.2-91.4	98.3-98.5	85-87	95.2-96.3
59	HuN25	2014	Swine	China	MF440653	90-90.1	97.9-98	83.7-84.2	92.9-93.9
60	HuN26	2017	Swine	China	MF440654	90.7-91	98.6-98.8	84.6-86.9	95.2-96.9
61	HuN27	2017	Swine	China	MF440655	91-91.2	98.4-98.5	84.3-86.8	94.2-95.9
62	HuN28	2017	Swine	China	MF440656	90.7-91	98.5-98.6	84.4-86.9	94.6-96.3
63	HuN29	2017	Swine	China	MF440657	89.5-89.6	97.7-97.9	82-85.2	92.3-93.9
64	HuN30	2017	Swine	China	MF440658	91.2-91.4	98.4-986	85.1-87.1	91.8-93.9
65	EF9-F	2016	Swine	Hungary	MN807773	-	-	62.6-64.5	60.6-61.6
66	SZ1M-F	2013	Swine	Hungary	MN807752	77.7	84.3-84.6	62.9-64.3	62-63
67	Bat/CAM/sap-p24	2013	Bat	Cameroon	KX644938				
68	Simian 1/strain 2383	2003	Monkey	USA	AY064708				
69	Avian- /TW90A	2004	Avian	Taiwan	NC006553				
70	Duck-TW90A	2007	Duck	Taiwan	AY563023				
71	WUHARV sapelo1/rhesus	2010	Monkey	USA	JX627573				
72	Human-poliovirus Sabin 1	1982	Human	USA	V01150				

Abbreviation: WGS, whole genome sequence; nt, nucleotide sequence identity; aa, amino acid identity; -, not available.
